# Curcumin and Osteosarcoma: Can Invertible Polymeric Micelles Help?

**DOI:** 10.3390/ma9070520

**Published:** 2016-06-27

**Authors:** Avudaiappan Maran, Michael J. Yaszemski, Ananiy Kohut, Andriy Voronov

**Affiliations:** 1 Department of Orthopedics, Mayo Clinic, 200 1st St SW, Rochester, MN 55905, USA; maran.avudai@mayo.edu (A.M.); yaszemski.michael@mayo.edu (M.J.Y.); 2 Department of Organic Chemistry, Lviv Polytechnic National University, vul. S. Bandery, 12, Lviv 79013, Ukraine; ananiy.kohut@gmail.com; 3 Department of Coatings and Polymeric Materials, North Dakota State University, NDSU Dept. 2760, P.O. Box 6050, Fargo, ND 58108-6050, USA

**Keywords:** invertible polymer micelles, micellar nanoassemblies, curcumin delivery, bone tumor treatment

## Abstract

Systematic review of experimental and clinical data on the use of curcumin in the treatment of osteosarcoma is presented. The current status of curcumin’s therapeutic potential against bone cancer is analyzed in regard to using polymeric micelles (including recently developed invertible, responsive, micelles) as a platform for curcumin delivery to treat osteosarcoma. The potential of micellar assemblies from responsive macromolecules in a controlled delivery of curcumin to osteosarcoma cells, and the release using a new inversion mechanism is revealed.

## 1. Introduction

Osteosarcoma is a common primary type of malignant bone tumor in children and young adults. As a very aggressive tumor, associated with poor prognosis, it is currently the sixth leading cancer in children under 15 years of age [1]. A second peak in the incidence of osteosarcoma occurs in the elderly, and is usually associated with underlying bone pathology, such as Paget’s disease, medullary infarct, or prior irradiation [2,3]. For children and young adults, osteosarcoma most frequently occurs in developing bones, and often results in metastases in the lungs and subsequent failure of the respiratory system [4,5]. Eventually, about 30% of patients with an osteosarcoma diagnosis develop lung metastases, which explains the fact that the overall prognosis is poor [2,6,7]. Current strategies for osteosarcoma treatment consist of radiation therapy and surgery, including adjuvant chemotherapy with various antitumor agents at high doses [2]. The standard clinical treatment consists of presurgical (neoadjuvant) chemotherapy, followed by surgical resection of the primary tumor after the second or third cycle of the year-long chemotherapy regimen. For the treatment of osteosarcoma, cisplatin, doxorubicin, adriamycin, methotrexate, ifosfamide, and etoposide are the most commonly used drugs [8,9].

However, the five-year survival rate for patients with metastatic osteosarcoma is still very low (20%) [2]. In addition, use of these drugs promotes toxicity and side effects in this aggressive cancer, as well as frequently causing chemoresistance [3,10,11,12,13].

### 1.1. Curcumin and Its Molecular Targets

Present in the spice turmeric, curcumin is derived from the rhizome of Curcuma longa and has been traditionally applied for the treatment of various diseases in Chinese and Indian medicine. This phytochemical has recently attracted the attention of clinicians as a powerful antioxidant with strong anti-inflammatory properties [14,15], and as a potential agent for anticancer therapy [16,17,18,19]. Although the bioavailability of curcumin is low, it provides a variety of beneficial effects in living organisms, including proliferation inhibition and an antitumor effect in various cancer cells [20,21]. There are many ongoing clinical trials worldwide that seek to understand the molecular mechanisms for curcumin activity in cells, as well as the biological effects of curcumin in both cell and animal model systems [22]. Although there are studies indicating that curcumin can be cytotoxic for normal cells [23,24,25,26,27], it has in general been shown that cancer cells are much more sensitive to curcumin than their normal counterparts [28]. Curcumin has not shown any adverse side effects in clinical trials to date, but its clinical application is currently limited, due to its low bioavailability and poor solubility in aqueous media (11 ng/mL). Even high amounts of oral administration (8 g/day) of curcumin result in extremely low serum levels (22–41 ng/mL) in humans [29]. One possible way to enhance the solubility of curcumin in aqueous media, and therefore its bioavailability, is by conjugation with polymers [30,31]. However, the therapeutic dose and the intracellular uptake are not fully established.

Cancer is well recognized as a disease that results from dysregulation of multiple cell signaling pathways [29]. Recent studies indicate that curcumin can interact with a diverse range of molecular targets and affect numerous molecular and biochemical cascades. To this end, transcription factors, cytokines, enzymes, kinases, receptors, and growth factors can be activated by pleiotropic molecules of curcumin [29]. In particular, a multitargeting ability can be a key property in accomplishing the therapeutic activity of curcumin against cancer.

Several signaling mechanisms have been suggested for the action of curcumin in osteosarcoma and other cell types, and the mechanism appears to be cell–type specific. To date, the antitumor activities of curcumin were shown in colorectal carcinoma [32], head and neck squamous cell carcinoma [33], and pancreatic cancer [34], as well as osteosarcoma [35]. It has been reported that expression of genes involved in invasion, metastasis, cell proliferation, apoptosis, and resistance to chemotherapy are modulated by curcumin [36,37,38,39,40]. However, the precise molecular mechanisms of the antitumor activity of curcumin in osteosarcoma still remain unclear.

### 1.2. Curcumin-Induced Apoptosis

Previously, curcumin activity in preventing osteosarcoma cell proliferation was attributed to drug-induced apoptosis [41,42,43]. A step-by-step examination carried out by Walters et al. using viability and apoptosis assays indicated that curcumin indeed induces apoptosis in osteosarcoma cells [35]. Authors investigated signaling pathways activated by curcumin and confirmed that curcumin-induced apoptosis in different osteosarcoma cell lines involves caspases in the process. Nevertheless, various signaling pathways can be affected by the presence of curcumin [44]. In addition, even a series of signaling events might be involved in curcumin-induced apoptosis of osteosarcoma cells, suggesting that additional signaling pathways might exist.

Recent studies indicate that, in particular, the proteins BAX and Bcl-2 and BAX can be involved in this process. Bcl-2 family proteins are key regulators of apoptosis, while BAX belongs to the pro-apoptotic group [45]. Antiapoptotic effects of Bcl-2 are exerted by mitochondrial cytochrome c release inhibition, while a pro-apoptotic effect of BAX is due to interacting with membrane pore proteins to increase release of cytochrome c [46,47]. Treatment of various osteosarcoma cells resulted in increased cellular levels of BAX and a decreasing cellular level of Bcl-2 [48,49]. Furthermore, the importance of curcumin-induced regulation of Bcl-2 and BAX in drug-induced apoptosis was demonstrated [50,51]. In a study by Walters et al. [35], treatment of osteosarcoma cell lines with curcumin resulted in decreased and increased cellular levels of Bcl-2 and BAX, respectively, indicating that these proteins are important regulators of drug-induced apoptosis in osteosarcoma.

### 1.3. Notch-1 Signaling Mechanism

Since the Notch signaling pathway plays important roles in human cancer, it has been suggested that specific agents targeting downregulation of Notch signaling can be useful for osteosarcoma treatment. The Notch plays a critical role in proliferation, apoptosis, differentiation, and stem cell maintenance [52], so the Notch signaling pathway may well contribute to osteosarcoma carcinogenesis. The obtained results showed that presence of curcumin leads to osteosarcoma cell death by activating the caspase-3 pathway and blocking cells successively in the G1/S and G2/M phases, as Lee et al. reported [53]. In work by Leow et al., [54] curcumin showed anti-invasive and antimetastatic effects towards intrinsic and extrinsic activation of the Wnt/b-catenin pathway, in part mediated by downregulating matrix metalloproteinase-9 (MMP-9). Previous reports about Notch-1 cross-talking with MMPs involved in metastasis and cancerous cell invasion have been published [55,56]. To this end, MMP expression was reduced by downregulation of Notch-1 [57,58,59]. Motivated by these observations, Li et al. investigated whether curcumin could inhibit the Notch signaling in osteosarcoma cells and how it might be related to various Notch-responsive genes [18]. Authors assessed the antitumor activity of curcumin using three different osteosarcoma cell lines (U2OS, SaOS-2, and MG-63), to see whether downregulation of Notch-1 is critical in curcumin-induced inhibition of proliferation and invasion in osteosarcoma cells. The results show that curcumin indeed exhibits inhibitory effects on cell proliferation and invasion via suppression of Notch-1 signaling, accompanied by downregulation of Hes-1, cyclin D1, MMP-2, and MMP-9. Moreover, Notch-1 specific downregulation via small-interfering RNA prior to curcumin treatment resulted in cell growth inhibition and invasion. It is clear that the novel mechanism of curcumin antitumor activity involves inactivation of the Notch-1 signaling pathway. Data obtained by Li et al. provided, for the first time, evidence for Notch-1 downregulation by curcumin as an effective mechanism for osteosarcoma treatment by this drug.

### 1.4. Effects of Curcumin on Normal Cells

There are differential effects of curcumin on normal cells, including lymphocytes, fibroblasts, thymocytes, mammary epithelial cells, and hepatocytes [60,61,62,63,64,65,66]. Several reasons have been suggested for the ability of curcumin to kill tumor cells but not normal cells, although this process is not fully understood. Kunwar et al. demonstrated that uptake of curcumin in tumor cells is higher than in normal cells [67]. Furthermore, uniform distribution of curcumin in the cell nucleus and membrane has been detected by this group using fluorescence spectroscopy. Another factor contributing to targeted activity of curcumin in tumor cells can be an enhanced sensitivity of cancer cells to this drug due to lower levels of glutathione, as reported by Syng-Ai et al. [61]. In addition, normal cells do not express active NF-κB as most cancer cells do, mediating their survival [68]. Curcumin can suppress gene products regulated by NF-κB and, in this way, suppress the proliferation and survival of tumor cells. In this regard, curcumin did not show cytotoxicity in normal rat hepatocytes, and no cell death has been observed [61]. Primary human cultures of fibroblasts were also found to be much less sensitive to lower doses of drugs [64], although curcumin caused fibroblasts’ apoptosis to be inhibited by the presence of antioxidants, as reported by Scharstuhl et al. [69]. Ravindran et al., in their review, have shown that, in general, curcumin can kill a broad variety of cancerous cells using diverse mechanisms, but not normal cells [28]; however, they mentioned the necessity of additional studies to gain the full benefits from curcumin in tumor treatment. At the same time, some studies demonstrated that normal osteoblast cells might be killed by curcumin as well. Chan et al. have shown that osteoblast apoptosis occurs at a low concentration of curcumin (up to 25 μM), and when the concentration is increased up to 200 μM, necrosis was detected [70]. In this regard, the critical information is which concentration of curcumin is not toxic to normal osteoblasts but kills different osteosarcoma cell lines. Such a study was recently carried out by Chang et al. [71]. In this work, the cytotoxicity of curcumin presented at different concentrations (5–100 μM) was compared between MG-63 osteosarcoma cells and normal human osteoblasts (HOBs). The obtained results show that MG-63 osteosarcoma cells are much more sensitive to the drug, with high viability of HOBs after 24 h of the experiment. To this end, it appears that curcumin indeed possesses a selective ability to kill osteosarcoma cells but not HOBs. Nevertheless, a proper curcumin delivery system as shown below (carrier) could help overcome its poor solubility in water and bioavailability, and enhance the potential of this drug against osteosarcoma.

## 2. Polymeric Micelles as a Platform for Drug Delivery

The poor solubility of many drugs and drug candidates is one of the main problems encountered when formulating clinically useful pharmaceuticals [72,73,74]. In the past two decades, over 90 chemotherapeutic drugs have been approved by the U.S. Food and Drug Administration for clinical applications, but the efficacy of these drugs has been dramatically hindered due to ineffective delivery systems. The premier drug delivery nanoparticle presently in use is the liposome [75]. The Doxil™ (Ben Venue Laboratories, Bedford, OH, USA) formulation, for example, contains a powerful and toxic chemotherapeutic—doxorubicin—incorporated into liposomes, and is approved for the treatment of Kaposi’s sarcoma, metastatic breast cancer, and recurrent ovarian cancer [76]. However, due to their hydrophilic interior, liposomes are best suited for the delivery of water-soluble agents and have highly limited loading capacity for poorly water-soluble drugs, as these are not readily entrapped within the hydrophobic bilayer of the liposomes [77]. In addition, most liposomes are larger than 100 nm in diameter, which hinders their transport into tumor tissues. Polymeric micellar assemblies with hydrophobic cores provide a carrier environment that can encapsulate poorly water-soluble hydrophobic anticancer drugs. The size of these assemblies is typically 10–100 nm and can be precisely controlled by varying the structure of the amphiphilic polymers by the choice of the length of the hydrophobic blocks. The available size range, compared to that of liposomes, prevents losses by renal filtration while allowing increased tumor penetration [78]. Controlled release of the therapeutic agent from the assemblies is also highly desirable to achieve the required level of cytotoxicity upon entry to the tumor site.

Amphiphilic block copolymers usually form polymeric micelles consisting of hydrophilic and hydrophobic fragments, due to their ability to self-assemble in aqueous solutions [79,80]. The driving force for this self-assembly is a hydrophobic interaction that leads to the formation of micellar polymeric architectures with a hydrophobic interior and a hydrophilic stabilizing exterior [81,82,83]. Micellization behavior, drug encapsulation, and the application of polymer micelles for drug delivery have attracted considerable recent interest [84,85]. Polymeric micelles can effectively accommodate (solubilize) hydrophobic substances by physically entrapping them in the hydrophobic interior [86,87,88,89]. Therefore, they are ideally suited for the delivery of insoluble drugs [90,91]. A number of recent studies have proposed block-copolymer assemblies as carriers for anticancer agents or other targeted therapeutics [92,93,94]. The hydrophobic micellar core is expected to regulate the retention and release of loaded drug molecules at an appropriate time scale [95,96]. The structures of amphiphilic block copolymers often include poly(ethylene oxide) (PEO) as the hydrophilic group of choice [97,98]. The length of the hydrophobic block is then chosen to be close to or lower than that of the PEO block. A variety of polymers have been used as hydrophobic interior-forming blocks [99,100,101]. Amphiphilic block copolymers made from PEO and poly(propylene oxide) (PPO) blocks (Pluronics) have also been evaluated in numerous studies as promising carrier systems for drug and gene delivery [102,103,104]. The noncovalent incorporation of the cargo into a hydrophobic block-copolymer core has been shown to increase the drug solubility and stability, as well as the circulation of the drug throughout the body [105]. Pluronics can exist in the form of unimers and of micelles encapsulating the solubilized drug; in fact, Pluronic unimers can themselves act as modifiers of biological responses by binding to the cell membrane, thereby altering membrane microviscosity and inhibiting the activity of select membrane proteins [106]. These multiple effects all emanate from the block copolymer’s interaction with the biomembrane. No specific copolymer sites exist for binding with specific cellular molecules, which implies a critical role for unspecific interactions between copolymers and biomembranes.

Different internalization pathways for polymeric unimers and aggregates have recently been demonstrated [107]. The Pluronic unimers enter cells rather selectively; a possible explanation for this phenomenon is that the more hydrophobic fragments of Pluronics recognize the cholesterol-rich domains of the cellular membrane. This selectivity may originate from differences in the binding energy of the polymer units in the block copolymer chain (the hydrophobic and hydrophilic fragments typically are distributed along the macromolecule). The Pluronics reversibly bind to the membrane, with an exchange occurring between unbound and membrane-bound macromolecules. Optimal binding species therefore can only be identified after multiple trials [108]. One recent study showed that poly(ethylene glycol)-polyester micelle–biomembrane interactions allowed the effective transfer of a core-loaded hydrophobic cargo to the plasma membrane, from which it could either endocytose or diffuse to targets or specific intracellular structures [109].

Much effort has been expended in the design of block copolymer platforms for drug delivery, but researchers still face challenges, such as low drug loading capacity, reduced targeting ability, short in vivo stability, and uncontrolled (non-sustained) drug release [110,111]. The future block–copolymer based delivery systems will require novelty in their macromolecular design, such as inclusion of targeting moieties that can enhance cellular uptake, particularly through receptor-mediated endocytosis, and the drug carriers will need stimulus-responsive features at the site of action. These systems belong to the second generation of polymer-based therapeutics, with properties that will improve their efficiency and reduce adverse side effects [112].

Using a solid dispersion method, micelles of poly(ethylene glycol)-*b*-poly(*ε*-caprolactone-*co*-p-dioxanone) were loaded with curcumin in a study by Song et al. [113]. The obtained results show that micellar loading capacity was about 12 wt %, and more than 95% of the curcumin had been successfully incorporated into the micelles. No burst release of the drug was observed, whereas 80 wt % of curcumin was released within 300 h of the experiment. Other work from this group reports synthesis of a poly(d,l-lactide-*co*-glycolide)-*b*-poly(ethylene glycol)-*b*-poly(d,l-lactide-*co-*glycolide) (PLGA-PEG-PLGA) triblock copolymer using PEG as macroinitiator of ring opening polymerization of d,l-lactide [114]. Critical micelle concentration of the PLGA-PEG-PLGA was determined to be 0.03 mg/mL. The synthesized triblock copolymer forms spherical micelles with a size of 30 nm in diameter, determined using transmission electron microscopy and dynamic light scattering measurements. Micellar loading capacity of PLGA-PEG-PLGA micelles was 4% *w*/*w*, and 70% of curcumin amount was incorporated into the micelles. The study did not report on the release experiment, but evaluated pharmacokinetic and biodistribution of curcumin-loaded micelles in vivo. The micelles with a drug were able to pass through the vascular endothelium, reach pulmonary alveoli, and accumulate in the lungs. Mixed micelles from Pluronics polymers F68 and P123 were formed for curcumin incorporation in a study by Zhao et al. [115]. F68/P123 mixed micelles showed a size of about 70 nm with a loading capacity of 7 wt % More than 85% of the curcumin was loaded into the micelles, which demonstrated the sustained release profile by releasing about a half of the loaded curcumin amount within three days of the experiment. Experiments on the molecular dynamics of curcumin interactions with Pluronics copolymers were carried out by Samantha et al. [116]. This study confirms that molecules of curcumin interact predominantly with the more hydrophobic poly(propylene oxide) fragments in Pluronics, while the more hydrophilic PEG sequences serve as a micellar outer part, so that curcumin becomes solvated in an aqueous environment. Using a solid dispersion method, micelles with an average diameter of about 30 nm from monomethyl poly(ethylene glycol)-poly(*ε*-caprolactone) (MPEG-PCL) were loaded with curcumin in study by Gong et al. [117,118]. The formed micelles were dispersible in water after freeze drying. The obtained results show that micellar loading capacity was about 15 wt %, and more than 99% of the curcumin had been incorporated into the micelles. In a dialysis experiment using phosphate buffered saline and 0.5% of Tween 80, about 60 wt % of curcumin was released within 14 days of the experiment. The cosolvent evaporation technique was used by Ma et al. to load curcumin in micelles from various PEO-PCL block copolymers [119]. The highest loading capacity was reported for PEO (fragment molecular weight, 5000)-PCL(fragment molecular weight 24,500) copolymer, although the most sustained release was observed for a block copolymer made from PEO_5000_ and PCL_13,000_. The authors observed faster release in the experiments where human serum albumin was presented, and explained the latter observation by a higher affinity of curcumin to the protein. Using a co-solvent evaporation method, curcumin as a therapeutic and pifitrin as a sensitizer were incorporated in micelles of miktoarm star polymers PEG_2_-PCL [120]. This work reported on a significant increase in aqueous solubility of curcumin and high efficiency in causing glioblastoma cell death. In the study by Yang et al. [121], curcumin was loaded into micelles of monomethyl poly(ethylene glycol)-poly(*ε*-caprolactone)-poly(trimethylene carbonate). The developed micelles had a particle size of 27.6  ±  0.7 nm, drug loading of 14.07%  ±  0.94%, encapsulation efficiency of 96.08%  ±  3.23% and were much more effective than free curcumin in inhibiting growth of CT26 colon tumor.

## 3. Invertible Polymer Micelles as a Safe Way to Treat Osteosarcoma

Micellar polymeric assemblies from amphiphilic invertible macromolecules represent a new polymer class, synthesized in our group to study polymer-mediated curcumin delivery to treat osteosarcoma [122,123,124,125,126,127,128]. The macromolecules of amphiphilic invertible polymers (AIPs) are made from a precisely controlled number of hydrophilic and hydrophobic short fragments with a well-defined length, alternately distributed in a macromolecular backbone. Incompatibility of these short macromolecular fragments results in microphase separation at smaller-length scale, as compared to block copolymers’ structure. The latter in turn enables a greater degree of control in micellar formation [122,123,124,125].

**Figure 1:**
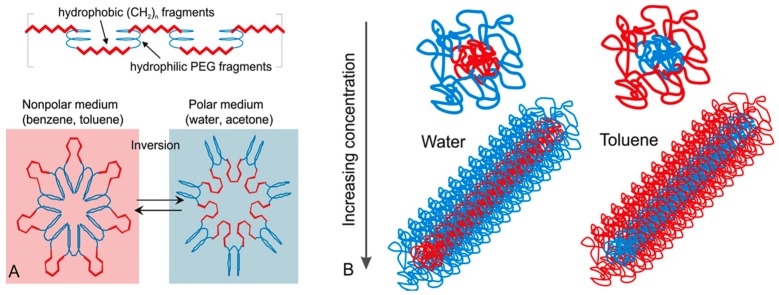
Amphiphilic invertible polymer and micelle structures (**A**) and micellar self-assembly (**B**) in polar and nonpolar solvents (reprinted with permission from [128]. Copyright 2011, American Chemical Society).

Our studies show that AIP macromolecules self-assemble into invertible micellar assemblies in response to changing polarity of the environment, polymer structure, and concentration (Figure 1) [122,123,124,125,126]. The alternated distribution of hydrophilic and lipophilic fragments in the macromolecular backbone facilitates additional control over the self-assembly. The varying hydrophilic-lipophilic balance (HLB) influences surface activity and self-assembly considerably [122,123,124,125]. Micellar polymer assemblies from AIPs can accommodate the lipophilic “guest” molecules in their interior and transfer the hydrophobic cargo from water to the less polar phase [123,124]. The amount of the transferred payload depends primarily on micellar loading capacity, but most of the loaded cargo molecules have been successfully delivered through the polar-non-polar interface by micellar assemblies [123,124]. To demonstrate the capability of the micellar assemblies to deliver lipophilic drugs, and release the payload using stimuli-responsive inversion of macromolecules, they were loaded with poorly water-soluble curcumin. Knowing that curcumin possesses a great potential in the treatment of diverse diseases, including cancer, but its clinical development is significantly hindered due to the aqueous instability of this drug, using micellar assemblies from AIPs was seen as a tool to address this issue by improving its aqueous solubility and bioavailability.

### 3.1. Self-Assembly of Invertible Macromolecules

**Figure 2:**
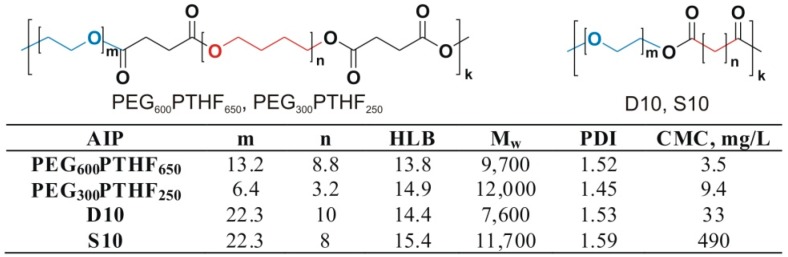
Chemical structure and characteristics of the representative AIPs. D10 is made from dodecanedioic acid and polyethylene glycol with an average Mn 1000 g/mol (PEG-1000). S10 is based on PEG-1000 and sebacic acid. The subscripted numbers indicate the average molecular weight of the copolymerized PEG and polytetrahydrofuran (PTHF) in the PEG-PTHF copolymers (reprinted with permission from [124], Copyright 2012, American Chemical Society).

AIPs possess a unique ability to undergo reversible changes in conformation in response to the changing polarity of the environment. Using poly(ethylene glycol) (PEG) as the hydrophilic constituent and aliphatic dicarboxylic (sebacic and dodecanedioic) acids as the hydrophobic constituent, or a combination of PEG and polytetrahydrofuran (PTHF) as the hydrophobic constituent, several AIP libraries have been synthesized [122,123,124,125,126,127]. The syntheses have been performed through a polycondensation that results in alternating invertible amphiphilic polyester macromolecules with various ratios of hydrophilic and hydrophobic constituents (a series of samples with different HLBs). The characteristics of four representative invertible polymers are shown in Figure 2.

The AIP macromolecules form micelles in different solvents and demonstrate a unique switching behavior when the solvent polarity is changed (Figure 1) [122,123,124,125,126,127]. In a polar medium, PEG (*blue*) forms the outer layer of the micellar architecture, and the hydrophobic fragments (*red*) are collapsed and screened in the micellar interior. The ability of the synthesized amphiphilic macromolecules to invert their conformation in response to changes in solvent polarity has been confirmed by viscosity measurements, ^1^H NMR spectroscopy, and small-angle neutron scattering (SANS) techniques [122,125].

A combined ^1^H NMR/SANS study has demonstrated that single AIP micelles aggregate and form supramolecular micellar assemblies by increasing the polymer concentration in an aqueous medium. The polymer micelles self-assemble and form nanostructures containing hydrophilic and lipophilic domains with AIP increasing concentrations at ambient temperature [122,123,124,125,126,127]. Changing the length and the ratio of the fragments in the amphiphilic macromolecule results in changes in the AIP surface activity in an aqueous solution. Even slight changes in the ratio of the sizes of the two (hydrophobic and/or hydrophilic) fragments have been conclusively shown to lead to remarkable differences in behavior of amphiphilic invertible macromolecules in response to concentration and polarity changes [122,125].

**Figure 3:**
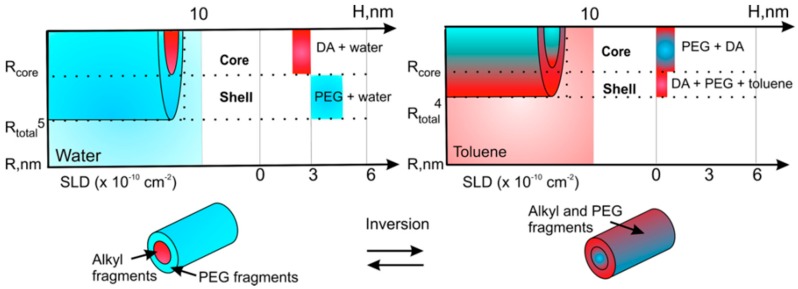
Scheme of the inversion for D3 (DA—dodecanedioc acid moiety) (reprinted with permission from [125], American Chemical Society).

To obtain detailed quantitative parameters of the formed nanostructures, SANS measurements and their fitting according to a core-shell cylindrical model were applied. The size and detailed composition of the micellar assemblies (including the effect of temperature on the size and composition of the assemblies) were measured in aqueous and toluene polymer solutions [125]. It was shown that AIP unimers self-assemble in aqueous solution predominantly through segregation of macromolecular fragments and the microphase separation. The longer –(CH_2_)_10_– fragments of dodecanedioic acid undergo stronger interactions compared to shorter –(CH_2_)_8_– sebacic acid fragments. The latter results in closer packing of fragments in the core and formation of smaller micellar assemblies [125]. To demonstrate the inversion mechanism of AIP micellar assemblies, scattering contrast and the known scattering length densities of the solvents and the polymer components have been used [125]. It was shown that PEG and the aliphatic dicarboxylic acid fragments of the AIP macromolecule indeed tend to replace each other in the core and shell of the assemblies in response to changes in the polarity of the environmental medium from polar to nonpolar (Figure 3). Furthermore, using various dyes, the ability of AIP micelles to solubilize otherwise insoluble substances, both in polar and nonpolar solvents, was demonstrated. The dyes were immediately extracted into the solvent phase, either water or toluene, in the presence of micellar assemblies, as indicated by the clear changes in the solution color [123].

By altering the molecular weight and ratio of the fragments in the macromolecule, solubility of AIPs in polar and nonpolar solvents can be adjusted. Highly hydrophobic drug molecules can be physically incorporated within the interior of the AIP micellar assemblies through hydrophobic interactions in an aqueous solution. This micellar inversion can be promising for rapid and controlled self-assembly in applications that require simultaneous utility in polar and nonpolar media, e.g., in drug delivery.

### 3.2. Micellar Assembly Mediated Curcumin Delivery to Osteosarcoma Cells

Upon adsorption onto the osteosarcoma cell surface, the AIP assemblies with incorporated curcumin molecules were expected to change their conformation and release their cargo (curcumin) for transport into the membrane. For this purpose in the experiments, micellar curcumin was prepared using 1% polymer solutions (Table 1). At this concentration, the AIP micelles are self-assembled into the assemblies, and the curcumin is solubilized through physical interactions with the polymer hydrophobic fragments of the micellar interior [124].

Table 1 shows the curcumin loading content for each micellar formulation that was investigated. For the four chosen polymers, the hydrodynamic diameters of the micellar assemblies vary between 4.7 ± 0.2 nm and 12 ± 0.6 nm. The hydrodynamic diameter size is larger for the loaded micellar assemblies in comparison to assemblies without curcumin, which confirms that the drug is incorporated into the micellar interior. Narrow micellar size distribution and small size of the assemblies indicate that they possess good physical properties to be considered as nanocarriers in delivery of drugs which are poorly water-soluble. The obtained results demonstrate that the highest amount of solubilized curcumin in the micellar assemblies is obtained with the most hydrophobic PEG_600_PTHF_650_ (HLB = 13.9). Using MTS (3-(4,5-dimethylthiazol-2-yl)-5-(3-carboxymethoxyphenyl)-2-(4-sulfophenyl)-2H-tetrazolium) assays for different concentrations of loaded assemblies, the effect of micellar assembly-mediated curcumin delivery on osteosarcoma cell survival was investigated.

**Figure 4:**
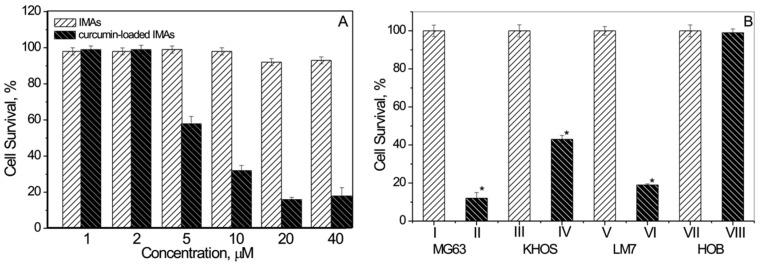
Osteosarcoma cell survival. MG63 osteosarcoma cells were treated with non-loaded micellar assemblies and micellar curcumin at different concentrations for 48 h (**A**); The effect of curcumin delivery on MG63 KHOS, LM7, and HOB cells at 72 h (**B**). Non-loaded micellar assemblies: I, III, V, VII; curcumin-loaded assemblies: II, IV, VI and VIII (from [126]).

The obtained results show that three human osteosarcoma cell lines: MG63 (treated with different concentrations of micellar curcumin, Figure 4A), KHOS, and LM7 (Figure 4B) demonstrated essentially reduced cell survival in the presence of micellar curcumin. Remarkably, the blank polymer micelles had no effect on the survival of normal cells, when tested at the same concentrations.

This indicates that the micellar assemblies effectively delivered curcumin and targeted three different bone cancer cells (MG63, KHOS, and LM7) but did not impact normal primary human osteoblast HOBs (Figure 4B).

These studies are comparable to the findings by Chang et al. [71]. It appears that a delivery system could help minimize the toxic effects observed by other investigators which show that curcumin at low concentration induces apoptosis and at high concentration induces necrosis in normal osteoblasts [70].

Thus, based on AIP, micellar–assembly delivered curcumin kills various osteosarcoma (cancer) cells by specifically targeting osteosarcoma cells and shows no effect on normal bone cells. The latter finding indicates that AIP–micellar assembly based delivery can be a powerful approach for the targeted delivery of poorly-soluble curcumin in bone cancer cells.

**Figure 5:**
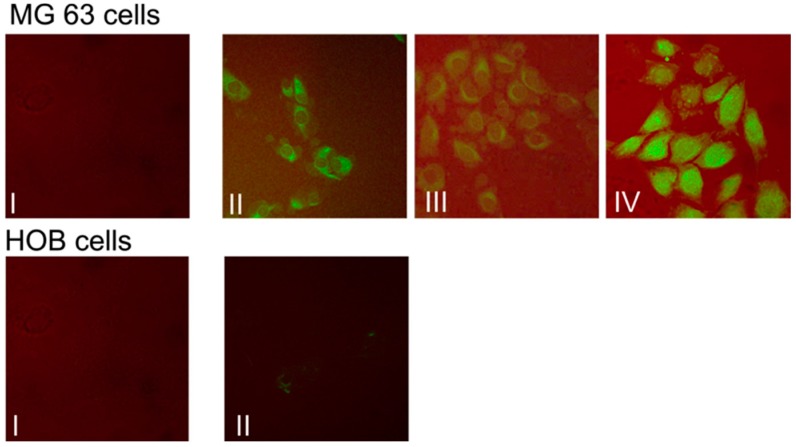
Micellar curcumin uptake in bone cells; 20 μM non-loaded assemblies (I) and micellar curcumin (II–IV) were exposed to MG63 osteosarcoma cells and normal human osteoblasts (HOB) for 30 min (I, II); 1 h (III), and 2 h (IV) (from [126]).

Differential effects of micellar curcumin in osteosarcoma and normal osteoblast cells were investigated next, by following the uptake of loaded and non-loaded micellar assemblies. When delivered by micellar assemblies, the curcumin was readily taken up, as confocal microscopy studies (Figure 5) confirms. The drug was seen in the cytoplasm within 30 min and could still be seen after two hours (Figure 5, top row). Based on confocal microscopy data, it was additionally confirmed that micellar assemblies are able to deliver the drug effectively and target bone cancer cells (MG63, KHOS, and LM7 cells), but not HOBs (Figure 5, bottom row).

The AIP micellar study shows the potential of assemblies from amphiphilic invertible macromolecules to be a promising platform for controlled delivery of poorly water-soluble curcumin to osteosarcoma cells, and to release the drug using a new and unexplored inversion mechanism. To reveal the nature of this novel and unique mechanism and its value in a treatment for bone cancer, further experiments are currently planned.

## 4. Conclusions

The drug release platform of the proposed polymeric carriers relies on switching (inversion) of the micellar architectures formed upon self-assembly in response to changing environmental polarity. Several stimulus-responsive strategies for drug release from polymeric micelles have been explored previously, including pH, temperature, and ultrasound, but it is expected that macromolecular inversion will prove more beneficial. It will facilitate carrier–cell interactions and enhance targeted drug release from the micellar assembly core into the cell membrane. However, the efficiency and robustness of this drug-delivery strategy can only be achieved when we fully understand the mechanisms of macromolecular inversion in response to environmental changes and all possible pathways of invertible micelle–cell interactions.

## Figures and Tables

**Table 1:** Physical properties of blank and curcumin-loaded micellar assemblies at 1% concentration.

AIP	Loading, wt %	Size, nm (Blank)	Size, nm (Loaded)	ζ-Potential, mV (Blank)	ζ-Potential, mV (Loaded)
**PEG_600_PTHF_650_**	10.3 ± 0.4	12.0 ± 0.2	17.5 ± 2.6	−24.2 ± 3.8	−18.2 ± 0.6
**PEG_300_PTHF_250_**	3.6 ± 0.3	12.4 ± 0.2	18.4 ±1.1	−43.0 ± 2.3	−42.6 ± 1.5
**D10**	1.9 ± 0.3	6.3 ± 0.3	7.8 ± 0.6	−10.3 ± 0.9	−7.4 ± 0.3
**S10**	0.14 ± 0.03	3.3 ± 0.5	3.6 ± 0.2	−7.1 ± 0.3	−6.7 ± 0.9

AIP—amphiphilic invertible polymer; PEG—poly(ethylene glycol); PTHF—polytetrahydrofuran.
